# High-resolution land use/cover forecasts for Switzerland in the 21st century

**DOI:** 10.1038/s41597-024-03055-z

**Published:** 2024-02-23

**Authors:** Luca Bütikofer, Antoine Adde, Davnah Urbach, Silvia Tobias, Matthias Huss, Antoine Guisan, Christophe Randin

**Affiliations:** 1 Centre alpien de phytogéographie CAP, Fondation Aubert, Route de l’Adray 27, CH-1938 Champex-Lac, Switzerland; 2 Department of Ecology and Evolution, University of Lausanne, Biophore, CH-1015 Ecublens, Switzerland; 3 Institute of Earth Surface Dynamics, University of Lausanne, Geopolis, Quartier Mouline, CH-1015 Lausanne, Switzerland; 4 Global Mountain Biodiversity Assessment, Institute of Plant Sciences, University of Bern, Altenbergrain 21, CH-3013 Bern, Switzerland; 5 Interdisciplinary Centre for Mountain Research (CIRM), University of Lausanne, Chemin de l’Institut 18, CH-1967 Bramois/Sion, Switzerland; 6 Swiss Federal Research Institute WSL, Zürcherstrasse 111, CH-8903 Birmensdorf, Switzerland; 7 Laboratory of Hydraulics, Hydrology and Glaciology (VAW), Swiss Federal Institute of Technology ETH, Hönggerbergring 26, CH-8093 Zürich, Switzerland

**Keywords:** Climate-change adaptation, Environmental impact

## Abstract

We present forecasts of land-use/land-cover (LULC) change for Switzerland for three time-steps in the 21^st^ century under the representative concentration pathways 4.5 and 8.5, and at 100-m spatial and 14-class thematic resolution. We modelled the spatial suitability for each LULC class with a neural network (NN) using > 200 predictors and accounting for climate and policy changes. We improved model performance by using a data augmentation algorithm that synthetically increased the number of cells of underrepresented classes, resulting in an overall quantity disagreement of 0.053 and allocation disagreement of 0.15, which indicate good prediction accuracy. These class-specific spatial suitability maps outputted by the NN were then merged in a single LULC map per time-step using the CLUE-S algorithm, accounting for LULC demand for the future and a set of LULC transition rules. As the first LULC forecast for Switzerland at a thematic resolution comparable to available LULC maps for the past, this product lends itself to applications in land-use planning, resource management, ecological and hydraulic modelling, habitat restoration and conservation.

## Background & Summary

From as early as 12,000 BP, anthropogenic activities like burning, hunting, cultivation, species translocation, deforestation and urbanisation have altered terrestrial landscapes around the globe^1^. The magnitude of these alterations is such that it became detectable in the geological record, which set the benchmark for the beginning of the so-called Anthropocene^2^—the first geological epoch dominated by anthropogenic activity. The main manifestations of the Anthropocene’s environmental changes are land-use/land-cover (LULC) changes^3,4^, which are brought about by the interplay of anthropogenic and environmental forces^5^ and are the main cause of biodiversity loss^6^. Two key tools for understanding LULC changes are LULC maps, which depict the spatial distribution of LULC classes, and LULC predictive models, which model their temporal dynamics^7^. LULC models can be used both as projection and as hypothesis-testing tools to gain insight onto the causes and consequences of LULC changes, and they can provide a computational basis to decision-making in land-use planning and policy^8^.

In Switzerland, landscapes have been sculpted by anthropogenic activities since the Bronze age, and are being further affected by climate change—especially in mountainous regions^9–11^. In the recent past, these changes have been monitored by the Swiss Federal Statistical Office, which has produced four iterations of aerial-survey-derived LULC maps (1979–85, 1992–97, 2004–09 and 2012–18)^12^. These maps classify the Swiss landscape into LULC classes at multiple thematic resolutions. The highest thematic resolution version (“NOAS04_72”) has 72 classes; additionally aggregations are made available at 27 (NOAS_04_27”) and 17 (NOAS04_17”) classes.

Efforts to optimise multiple goals across policy sectors—e.g. agriculture, forestry, industry, urban development, tourism, and nature conservation—often result in competition for limited land resources^6,13^. As a consequence, LULC maps for the future as well as a LULC model able to inform alternative management scenarios are critically needed to facilitate landscape-related decision making. Gerecke *et al*.^14^ provided projections of LULC transitions for four classes (“Built-up area”, “Lowland agriculture”, “Alpine agriculture”, and “Forest”) for the 2009–2081 period in the context of modelling trade-offs among landscape services. However, LULC forecasts with a thematic resolution that matches LULC maps already available for the past is still missing. We filled this gap with 100-m geographic projections of LULC change for three time-steps at 30-year intervals in the 21^st^ century (2020–2049, 2045–2074, and 2070–2099) under the Representative Concentration Pathways (RCP) 4.5 and 8.5. For this, we used a pattern-based approach^15^ structured in four steps: (1) a Environmental Suitability module^16–18^; (2) a Demand module^19–21^; (3) a Transitions module^19^; and (4) an Allocation module^22^. Additionally, glacier retreat was accounted for by including the mechanistic projections of Steffen *et al*.^23^. The resulting LULC maps have the same thematic resolution of 17 classes that can also be found in the LULC maps of the Swiss Federal Statistical Office^24^.

This dataset^25^ represents the first set of LULC projections made at the same thematic and spatial resolution of available LULC maps for Switzerland. This compatibility with past Swiss LULC products ensures its suitability for several applications including long-term land-use planning support and resource management, prioritisation of conservation and habitat restoration, hydraulic modelling and water usage plans, dynamic global vegetation models, and species distribution models^26–28^. The dataset was mandated by the Swiss Federal Office for the Environment, which aims at integrating our results in their future environmental policies.

## Methods

**Fig. 1:**
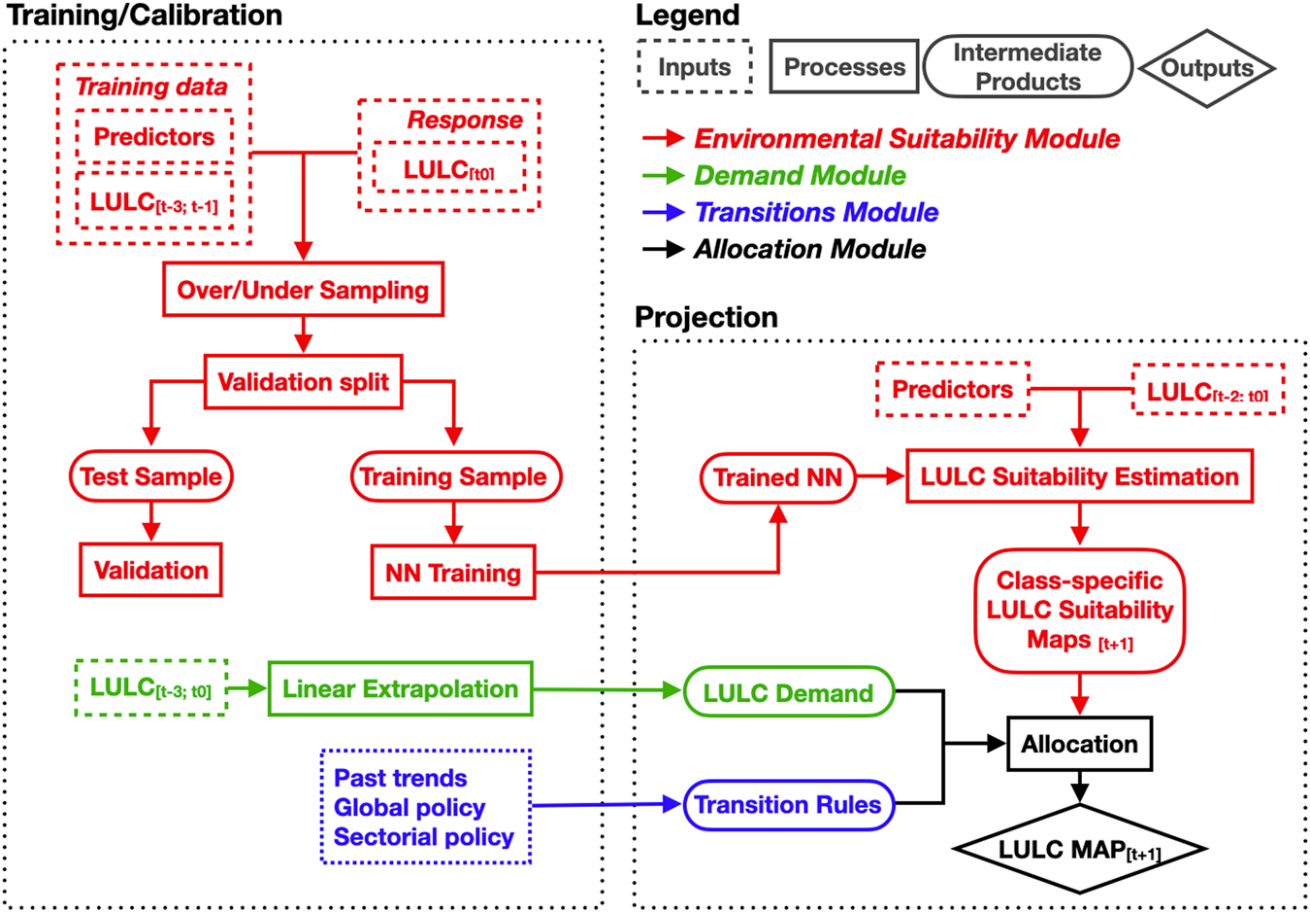
Four-steps (red, green, blue and black) workflow scheme for the LULC predictive model used to produce the LULC forecasts for Switzerland. The NN of the Environmental Suitability module (red) is trained with LULC maps for 1979-85, 1992-97, and 2004-09 and other predictors as independent variables, and the LULC map for 2012-18 as the response variable. Once trained, the NN is iteratively fed LULC maps of three consecutive time-step to predict the next one. Its output—a set of LULC suitability maps (one map per LULC class) showing the likelihood of finding each LULC class—is used in combination with that of the Demand (green) and Transition modules (blue) to allocate (CLUE-S algorithm) each 100×100 m cell in Switzerland to a single LULC class (Allocation module, black)—effectively returning a standard LULC map (final diamond box).

The four-step workflow (Fig. 1) used in preparing the LULC forecasts largely matches the typical architecture of inductive LULC change models^29,30^. The first step is the Environmental Suitability module, which provides information on where each LULC class is likely to occur in the study area at each time-step (Fig. 1, red pipeline). This is done in two versions, one with climate data derived for RCP 4.5, and one for RCP 8.5. The second step is the Demand module, which quantifies the prevalence of each LULC class for each time-step (i.e. the proportion of land covered by that class; Fig. 1, green pipeline); here no distinction between RCPs is made. The third step is the Transitions module (Fig. 1, blue pipeline), which defines which LULC transitions are allowed and which are prevented. Finally, the fourth step is the Allocation module, which combines the information provided by the previous three modules into a single LULC map for each time-step (Fig. 1, black pipeline); here two versions reflecting different RCPs are produced.

The Environmental Suitability module would in principle be sufficient to produce LULC forecasts. However, these forecasts would be entirely based on information contained in the module inputs. The Demand, Transitions, and Allocation modules—in addition to preventing possible inaccuracies of the Environmental Suitability module—allow the refinement of the output of the Environmental Suitability module by accounting for foreseeable changes in future LULC drivers, such as planned changes in policy. Different LULC forecasts can be obtained by varying the parameters of Demand and Transition modules, which are then integrated in the final forecast by the Allocation module. Our forecasts are the result of a single scenario that combines “baseline” future projections of data-driven trends from the past, with “modified” future projections whenever currently available sectorial policies clearly define future deviations from the baseline forecasts. These “modified” projections can be parameterised in the Demand module, the Transition module, or both. For this reason, in the section titled “Scenario description”, we describe our modifications to the baseline projections, and link them to the steps in the workflow that realise them. Although the single scenario we produced is made available in two versions reflecting different RCPs, RCPs only affected the climate data fed to the Environmental Suitability module, and not in the Demand, nor in the Transitions modules.

We describe our four-step workflow in details in the next four sections.

### Step 1: Environmental suitability module

To produce the past LULC raster maps we downloaded the GeoStat data (available in CSV format at the direct download link https://dam-api.bfs.admin.ch/hub/api/dam/assets/25885691/master) and extracted the easting, northing and LULC values for the study period (columns E, N, and AS85_72 till AS18_72). These maps are derived from aerial images at a native resolution ranging from 25 to 50 cm. Classes are attributed to points on a 100 m grid but the point’s neighbourhoods are considered in the process (e.g. class “Trees” is attributed to groups of at least three trees no more than 25 m apart). We converted the point grid to raster format by attributing the points’ values to 100 m × 100 m raster cells centred around each point.

The Environmental Suitability module aims at computing the suitability of each pixel for one of the 17 LULC classes of NOAS04_17 at each time-step. However, of these 17 classes, projections are made for only 14 of them, while the remaining three classes (“Lakes”, “Rivers”, and “Transportation”) remain static throughout all computations (with the exception of new Alpine lakes formed as a consequence of deglaciation^23^) either because they would have been too computationally intensive for reliable modelling, or because their expected change in spatial extent and position during the modelled period was considered negligible. These static classes have eventually been replaced by their counterpart from the highest thematic resolution NOAS04_72 in the final maps, bringing the thematic resolution of the final maps to 26 classes (i.e. the static class “Lakes” from NOAS04_17 remains a single class in NOAS04_72, while class “Rivers” from NOAS04_17 was replaced by the classes “Rivers” and “Flood protection structures” from NOAS04_72, and class “Transportation” form NOAS04_17 was replaced by the nine counterpart classes from NOAS04_72, see Table 1 for details).

**Table 1:** LULC classes in the LULC map forecasts.

Land-use classes					
Modelled		Static			
17-Classes Name	17-classes No.	17-Classes Name	17-classes No.	72-Classes Name	72-Classes No.
Industry	1	Transportation	15	Motorways	15
Building	2			Green motorway environs	16
Special urban	3			Roads and paths	17
Urban green	4			Green road environments	18
Horticulture	5			Parking areas	19
Arable	6			Sealed railway areas	20
Grassland	7			Green railway environments	21
Alpine grassland	8			Airports	22
Forest	9			Airfields, green airport environments	23
Brush	10	Lakes	16	Lakes	24
Trees	11	Rivers	17	Rivers	25
Unproductive vegetation	12			Flood protection structures	26
Bare land	13				
Glacier	14				

“Modelled” indicates the classes predicted by the NN. “Static” classes are kept unchanged during the modelling period (2020–2099). “No.” refers to the integer with which classes are encoded in the raster files. 17-Classes refers to the “NOAS04_17” version of the Swiss Federal Statistical Office, while 72-Classes to “NOAS04_72”.

The Environmental Suitability module is based on a feedforward Neural Network (NN) that predicts the suitability of the landscape for the 14 non-static LULC classes based on predictor variables. The output of the NN is a set of suitability maps for each land-use class at the next time-step. Spatially explicit predictor variables used to train the NN include LULC maps for three past time-steps (1979–85, 1992–97, 2004–09) as well as a range of climatic, ecological, and socio-economic predictors including the dominant land-use class in a 5 km × 5 km neighbourhood, five major river basins, proportion of each land-use class in a 5 km × 5 km neighbourhood, noise from transportation, cantons (i.e. Swiss administrative regions, used to account for differences in landcover management arising from variations in cantonal policies), distance to roads of 5 classes based on size, 19 climatic variables, distance to urban areas of 3 classes based on size, 10 soil variables, population density, bed-rock type, population density in a 9 km × 9 km neighbourhood, 14 hydrology variables, elevation, distance to lakes of 3 classes based on size, slope, and distance to rivers of 3 classes based on size (see Supplementary Table 1 for a complete list of predictors). All these variables were collated in a single dataset. The response variable during training was the current (2012–18) LULC map. As we wanted the NN to learn LULC patterns that span more than only a single or double time-step, we performed training with three past time-steps as predictor variables and the current time-step as response variable rather than using only one or two past time-steps as predictors and the following time-step as response.

All predictors were converted in 100 m × 100 m resolution raster files in the CH1903 + /LV95 (EPSG:2056) projected Swiss coordinate system and resampled to match the cell coordinates of LULC rasters. All spatial processing was carried out in R^31^. The total number of predictors was reduced by removing correlated variables among the continuous ones with R package “covsel”^32^; all categorical variables (marked as “categorical” in Supplementary Table 1) were one-hot encoded^33^ (i.e. each categorical variable was transformed into as many variables as its number of classes, each new variable reporting the presence (value of 1) and the absence (value of 0) of its corresponding class) and were not passed through the variable selection algorithm as it handles continuous variables only. The detection of correlated variables was done only on the training dataset and assumed constant throughout the modelling period because the NN requires the same variables at each time-step. The final number of predictor variables after selection and one-hot encoding entering the NN was 242 (i.e. the number of input nodes).

The NN architecture was selected by iteratively evaluating its cross-validation accuracy (validation split of 0.2) and the value of the loss function as well as the evolution during training of these two statistics (Fig. 3). The best performing architecture was a 242 neurons input layer, followed by five densely connected hidden layers with Rectified Linear Unit (ReLU) activation function^34^, separated by four dropout layers (0.2 dropout rate), followed by a 14 neurons output layer (one for each LULC class) with a softmax activation function^35^. NN training and projection was carried out with the Keras interface for TensorFlow on Python^36^.

NN training was carried out on a sample of 775936 cells. A balanced sampling approach^37^ was adopted to compensate for the relative scarcity of cells that change LULC at any time-step. Thus all changing cells were included in the sample together with an equal number of non-changing cells; this way the NN would not just ignore the much rarer “changing cells”. Since some LULC classes (e.g. urban classes) were highly underrepresented, all classes except the most prevalent one were oversampled with the Synthetic Minority Over-sampling TEchnique-Nominal Continuous (SMOTE-NC) algorithm^38^ provided in R package “RSBID” to a sample size equivalent to that of the most prevalent class; this way the NN would not just ignore the rarer classes. Oversampling was conducted only for the changing subset of cells. This is because the number of cells of the most prevalent class among the changing subset was still smaller than the number of cells for the least prevalent class among the non-changing subset. Summarising, the final training sample contained an equal number of changing and non-changing cells as well as an equal number of cells for each class. All features were centred and scaled by subtracting the mean and dividing by the standard deviation.

For each future time-step t + 1, the LULC predictor variables (LULC maps and LULC neighbourhood variables) derived from the past three time-steps (t-2, t-1, and t0) were updated (e.g. for the first future projection t + 1, the LULC maps for 1992–97, 2004–09 and 2012–18 were used as predictor variables to predict the LULC map for 2020–49) together with the climatic variables, while all other predictors were left unchanged. Two versions of the predictive step were produced whereby the climate variables were derived for different RCPs (RCP 4.5, and RCP 8.5). The softmax values of each output node for all raster cells resulted in suitability maps for each LULC class. These 14 maps (one for each predicted class) were combined together with the outputs of the Demand module and Transitions module in the Allocation module to finalise the LULC forecasting.

### Step 2: Demand module

The aim of the Demand module is to allow for direct control over the prevalence of each LULC class in the LULC map forecasts. We derived quantitative estimates of LULC prevalence for the future (i.e. LULC demand) by first computing the prevalence of each LULC class from the observed LULC maps (1979–85, 1992–97, 2004–09 and 2012–18), and then performing a linear extrapolation for the future three time-steps (2020–2049, 2045–2074 and 2070–2099) with the R function “approxExtrap” from the “Himsc” package, which computes future prevalences by projecting the prevalence slope observed between 2045–2074 and 2070–2099.

To account for future sectorial policy changes, we maintained constant the prevalence of urban classes (“Industry”, “Building”, “Special urban”, “Urban green”) and “Orchard” while forecasting the 2045–74 and 2070–99 time-steps. This is because urban expansion in Switzerland is going to be limited by the Swiss federal act on spatial planning (Spatial Planning Act^39^) currently in the process of being implemented by cantons and municipalities. Additionally, orchards are increasingly preserved thanks to the national strategies for the preservation of arable land, biodiversity, and climate change mitigation (Swiss Biodiversity Strategy and Action Plan^40^; Sectorial Plan of Cropland Protection^41^; Federal council strategy for adaptation to climate change in Switzerland^42^).

The Demand module does not vary according to RCPs. Instead, both RCP 4.5 and RCP 8.5 share the same LULC demand.

### Step 3: Transitions module

The goal of the Transitions module is to define transition rules. These determine which land-use class can be converted into which other ones. Certain transitions are prohibited because of their unlikeliness (e.g. conversion of urban into glacier), their economic unviability (e.g. conversion of urban areas into forests would waste the initial development investment^22^), or because of policy changes. For instance, future urbanisation is restricted by the Spatial Planning Act but currently scheduled development projects will be allowed to proceed. Therefore, to allow for some leeway in the implementation of the Spatial Planning Act, we permitted urban expansion for the first time-step and prevented it afterwards.

To allow for different rules at different times, two transition matrices were produced (Supplementary Tables 2, 3), one for the first (till 2020–49) and one for the second and third (2045–74 and 2070–99) time-steps. Transition rules are fed to the Allocation module in the form of a matrix with the current land-use classes as rows and the future land-use classes as columns. Allowed and forbidden transitions are marked with 1 and 0, respectively.

### Step 4: Allocation module

The Allocation module combines the outputs of the Environmental Suitability module, the Demand module, and the Transition module into a single LULC map per time-step (i.e. 2020–2049, 2045–74 2074, and 2070–2099, each in two versions reflecting different RCPs), thus allowing to implement deviations from the data-driven, “baseline” projections produced by the NN. We performed allocation with the Land Use and its Effects at Small regional extent (CLUE-S) algorithm^17^. In brief, CLUE-S uses LULC demand to allocate the requested number of map cells for each class at each time-step. It allocates each class starting from the cells with the highest suitability score (NN output node activation), then gradually proceeds by filling in cells with progressively lower suitability. Competition in cells with high suitability for multiple classes is addressed by balancing environmental suitability with LULC demand. This is all done while accounting for transition rules, which define which LULC class transitions are allowed.

We used the CLUE-S model as implemented in R package “lulcc”^29^. However, since the “lulcc” package does not support NN classifiers, we used the hidden function “.clues” in isolation from the “allocate” function (see supplementary material for the accuracy of the model in delivering the exact LULC demand). For each time-step, the Allocation module combines the environmental suitability maps produced by the NN into a single LULC map by allocating each cell to a single class according to its suitability for it as expressed as output node activation, the feasibility of the transition according to the predicted LULC demand for the class, and the transition rules. The two RCP versions produced by the Environmental Suitability module are treated in the same way in the Allocation module, thus generating two versions of the LULC map which reflect the two RCPs.

After the allocation step was completed, to accurately represent the dynamics of glacier retreat and the formation of new lakes from their thawing, we replaced all cells classified by our model as “Glacier” with “Bare rock” and then overlaid the final LULC map with the output of a mechanistic glacier and glacial lake model^23^. These steps results in a different prevalence of the “Glacier” and “Bare rock” classes than what required by the Demand module. However, the mechanistic glacier model we used is much more sophisticated than the linear extrapolation we used to infer “Glacier” and “Bare rock” prevalences, thus greatly adding to the realism of the LULC forecast.

### Scenario description

Both the LULC demand and the transition rules are key in steering the simulation of future Swiss landscapes. These were set to produce a “baseline” scenario reflecting minimum changes to the past LULC change dynamics (with the only departures from this trend accounting for the evolution of urban planning and biodiversity conservation policies, see “Step 2: Demand module”). To facilitate the understanding of their influence, we list here a few important characteristics of the simulation that are dictated by LULC demand and transition rules:

1. No class can take over land occupied by urban classes as too many resources were spent in the past to convert this land into urban areas (parameterised in the Transition module).
2. Urban expansion is prevented starting from 2045–2074 as a consequence of the implementation of the Spatial Planning Act and the expected population growth (parameterised in the Transition and Demand modules).
3. “Arable” and “Grassland” can be urbanised, but only during the first time-step (2020–2049). The two classes are always allowed to transition into one-another to reflect both crop rotation in mixed agricultural systems and permanent conversions. These two classes are also allowed to take over “Forest”, “Brush”, and “Unproductive vegetation”, but only to the extent that satisfies the baseline LULC demand (parameterised in the Transition and Demand modules).
4. “Alpine grassland” can take over “Forest”, “Brush”, “Trees”, and “Unproductive vegetation” to simulate the process of clearing forested areas to preserve valuable agricultural or man-made landscapes as allowed by the 2012 amendment on the federal law on forestry^39^. The natural vegetation dynamics following Alpine pasture abandonment is simulated by the sequence “Alpine grassland” to “Brush” (or “Trees”), to “Forest”. Therefore, transitions along this sequence are only allowed in the forest-ward direction (i.e. “Forest” cannot be taken over by “Trees” or “Brush”) (parameterised in the Transition module).
5. Forest expansion on built and cultivated land is prevented, but it is allowed on “Alpine grassland” to simulate Alpine pastures abandonment. It is also allowed over “Brush”, “Trees”, and “Unproductive vegetation” to simulate the natural vegetation dynamics. As the rate of future abandonment of Alpine pastures is dictated solely by extrapolation from past trends, the upwards shift of the treeline in Alpine environments is controlled primarily by land management rather than climate change—which would otherwise show a much faster colonisation of Alpine grasslands by forests^43^ (parameterised in the Transition and Demand modules).
6. As the land-use classes “Trees”, “Brush” and “Unproductive vegetation” can be ambiguous at times, we allowed these classes to transition into one-another. However, “Trees” are the only forested class that is allowed to take over “Grassland” to simulate the formation of new hedges. These can be planted for both agricultural (windbreakers) and biodiversity conservation (as habitats) purposes (parameterised in the Transition module).
7. Orchards (i.e. “Horticulture” class) are maintained to reflect efforts in biodiversity conservation and carbon sequestration. This class can take over land occupied by “Arable”, “Grassland”, “Trees” or “Unproductive vegetation”. However, transitions to “Horticulture” are only allowed to the extent of replacing losses as this class has been declining in the past and we have simulated its conservation in the future (parameterised in the Transition and Demand modules).
8. The natural glacier retreat dynamic is simulated by the sequence “Glacier” to “Bare land” to “Unproductive vegetation”. Transitions among these classes are only allowed in the direction of “Unproductive vegetation”—which is the only class that can take over “Bare land”. “Unproductive vegetation” can then become “Trees”, “Brush”, “Forest”—simulating forestation—or “Alpine grassland” simulating the formation of new high-elevation pastures (parameterised in the Transition module).

## Data Records

We deposited our LULC maps^25^ on EnviDat (10.16904/envidat.458). The dataset is composed of a compressed (.zip) file containing three georeferenced raster files in the GeoTIFF format with embedded coordinate reference system; a table (comma separated values,.csv) encoding a colour palette for plotting; and the code used to generate the LULC forecasts (see Code Availability section). GeoTIFF file names include the time-step and the RCP (e.g. “LULC_2020_2049_RCP45.tiff”). Each raster file has 3484 × 2207 square pixels (i.e. 7.7 megapixels) and is projected in the Swiss coordinate system CH1903 + /LV95 (EPSG:2056). Cell values are integers from 1 to 26 representing the LULC classes as listed in Table 1.

**Fig. 2:**
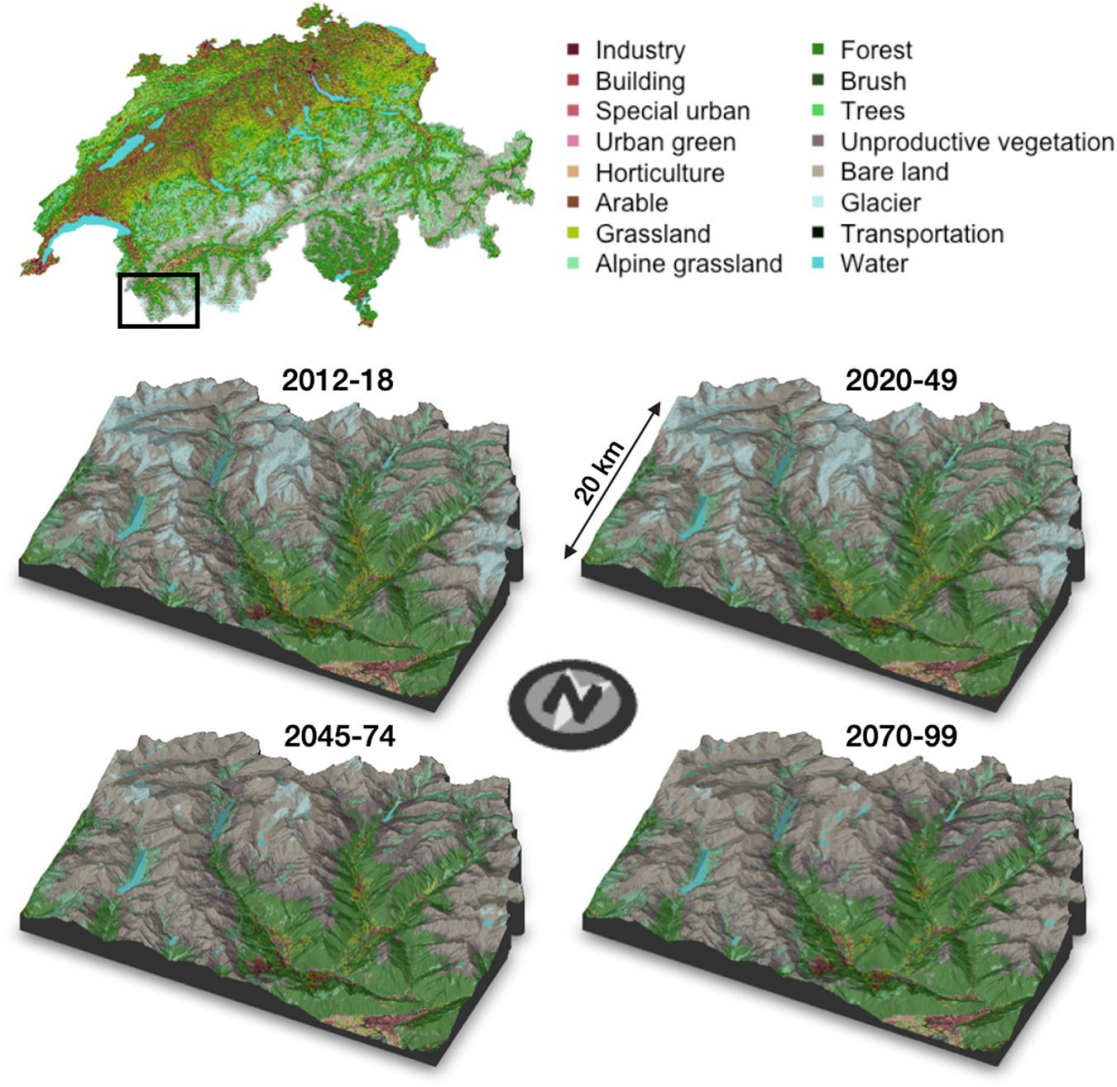
LULC maps for Switzerland. Top: the whole country for 2012-18. Bottom: enlargements to a sample Alpine region (Entremont district) for 2012-18, 2020-49, 2045-74, and 2070-99.

The colour palette table contains the HEX colour codes for each class to reproduce the maps in Fig. 2. For the sake of clarity, this colour palette aggregates all transportation and freshwater classes together, effectively plotting the 17 classes of “NOAS04_17” (see Table 1).

## Technical Validation

We validated the performance of (1) the NN and (2) the allocation algorithm separately. (1) When validating the NN we used it as a classifier and attributed each data point (i.e. pixel) to the output node with the highest activation (i.e. the most suitable LULC class). The NN’s performance therefore reflects the capacity of the model to forecast future LULC based purely on past trends.

In our normal workflow, the allocation algorithm—by integrating Demand and Transitions modules—allowed us to modify the output of the NN in order to account for future LULC change drivers that the NN had no access to during training (e.g. future implementation of the Spatial Planning Act). Since the whole point of introducing the Demand and Transitions modules in our workflow was to modify their parameterisation when projecting to the future, validating the performance of the whole workflow for the prediction of current LULC would provide no information on the forecast’s accuracy.

**Fig. 3:**
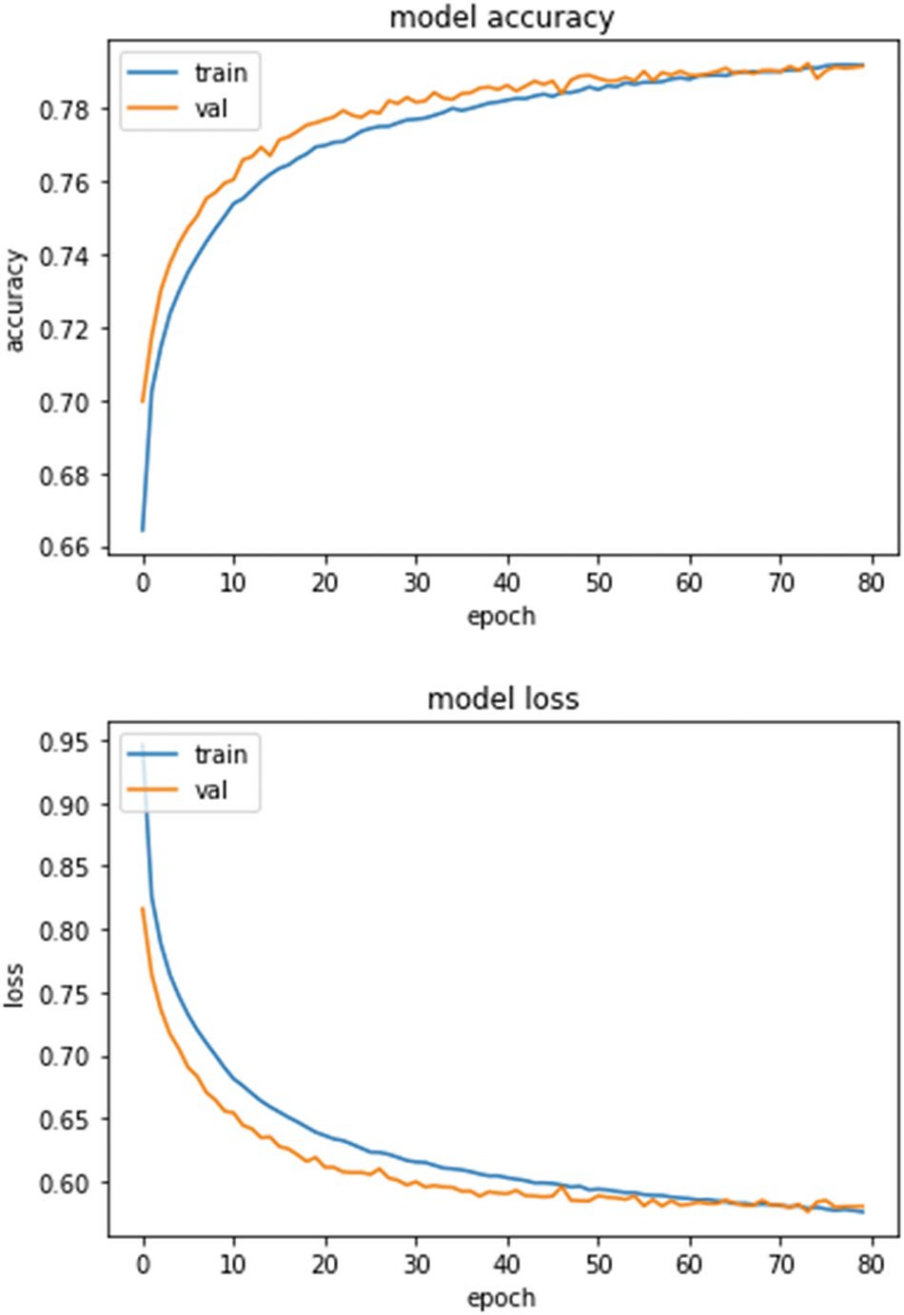
Neural network training summary of cross-validation accuracy (top) and loss function (bottom) values (both statistics are unit-less) against epochs.

The evolution of the NN accuracy and loss function during training (Fig. 3) shows that a plateau was reached by the 80^th^ training epoch showing that peak performance was reached. The convergence of the test and training lines indicates that the NN generalises well.

The dataset prepared to train the NN was split for cross validation (validation split of 0.2) and only 80% of it was actually used for training while the remaining 20% was kept aside for testing. Since the test split was extracted from the NN training data, it contained an equal number of changing and static cells, as well as the same number of cells from all land-use classes. This ensured that testing was not biased by an overwhelming proportion of easily learnable patterns like static cells or very consistent change patterns. The accuracy of the trained NN was tested on the test subset by computing overall accuracy rate (0.79, with a 95% confidence interval ranging from 0.7912 to 0.7948), quantity disagreement (0.053) and allocation disagreement (0.154)^44^, as well as class-specific statistics of Sensitivity (true positive rate), Specificity (true negative rate), and Balanced Accuracy (i.e. mean of sensitivity and specificity) (Table 2).

**Table 2:** Class-specific statistics of cross-validation classification performance of the Neural Network.

	Classes													
	1	2	3	4	5	6	7	8	9	10	11	12	13	14
Sensitivity	0.96	0.90	0.65	0.64	0.84	0.91	0.83	0.76	0.97	0.89	0.79	0.46	0.85	0.66
Specificity	0.99	0.99	0.97	0.98	0.98	1.00	0.98	0.99	1.00	0.98	0.96	0.98	0.98	0.98
Balanced Accuracy	0.97	0.95	0.81	0.81	0.91	0.95	0.91	0.88	0.98	0.93	0.88	0.72	0.91	0.82

The average class-wise balanced accuracy is 0.89, with the lowest value of 0.72 for “Unproductive vegetation” and a maximum of 0.98 for “Forest”. The lowest performance for the “Unproductive vegetation” class may be explained by its wide definition which encompasses visually diverse landscapes such as bushes, ski slopes, un-grazed grassland, and wetlands. The class-specific sensitivity value (0.46, specificity is 0.98) is responsible for the low score and probably indicates that the NN classified “Unproductive vegetation” cells as “Grassland”, “Forest”, or “Brush”. Please note that these comparisons are between our model results and the GeoStat LULC products. Since the GeoStat classification is not free from errors and we used these maps as input variables, their error will also be present in our results (unfortunately we could not find data on the accuracy of the original GeoStat classification).

**Fig. 4:**
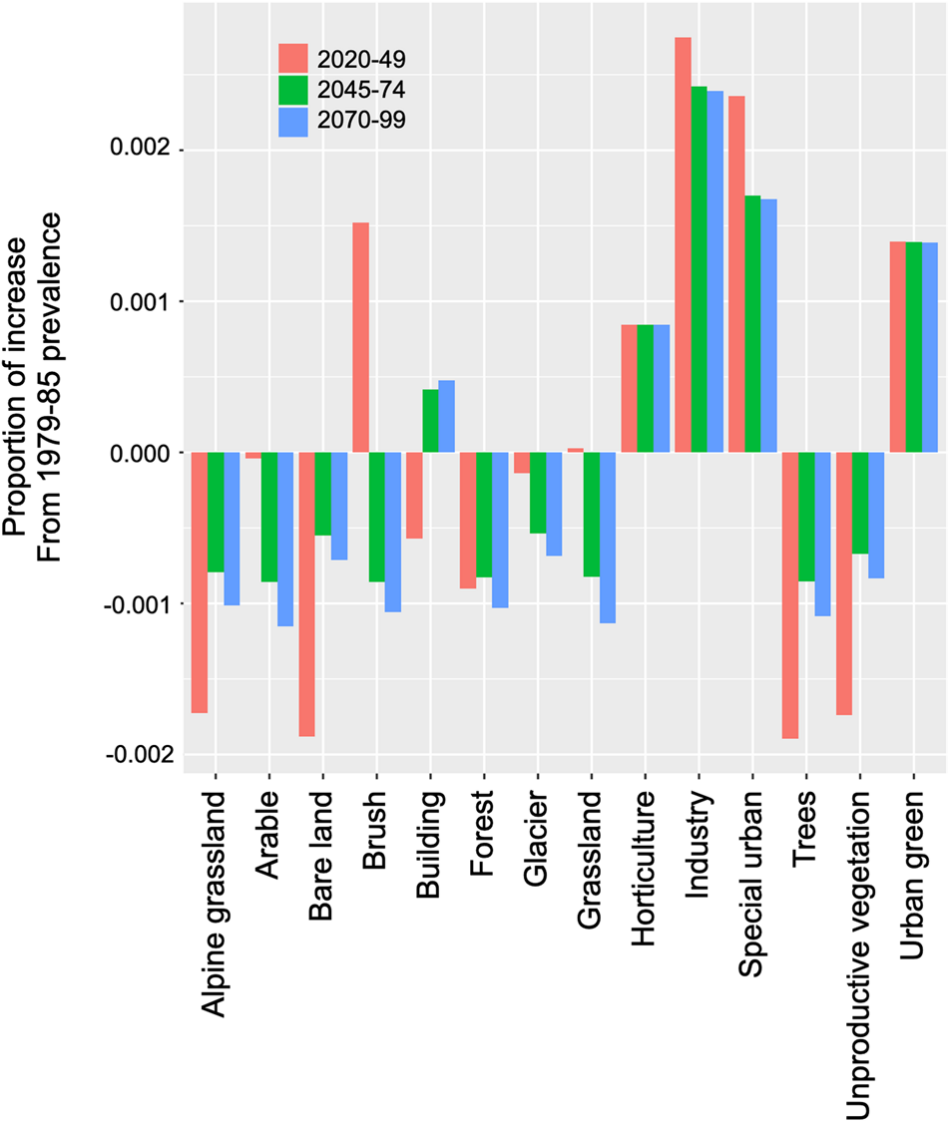
Comparison of LULC demand versus the LULC prevalence returned by the allocation. Bar values indicate the difference between the requested increase in prevalence with reference to the 1979-85 time-step, and the obtained one after allocation (e.g. if a class is required to increase its prevalence from 1979-85 by 0.3 (30% more prevalent in the study area) and the allocation returns an increase of 0.4 the bar will have a value of 0.1).

(2) As multiple LULC classes may have high suitability for the same cell, the allocation algorithm may not succeed in allocating the exact requested prevalence to each class. After the allocation completed, we measured the difference among the requested and obtained class prevalences (Fig. 4). Results show that most differences are very small—less than 0.2% difference between the requested and obtained increase, indicating that CLUE-S managed our allocation requests successfully.

## Usage Notes

Below we provided a sample R code to read and display the LULC map for the 2020–49 time-step with R. However, our files can be easily read with other geographic information systems like ArcGIS and QGIS.


library(terra)# Read mapr < - rast("LULC_2020_2049_RCP45.tiff")# Aggregate transportation and freshwater classesr[r > 14 & r < 24] < - 15r[r > 15] < - 16# Read colour palettecols < - read.csv("ColourPalette.csv")# Plot mapplot(r,   type = "classes",   col = cols$Colour,   levels = cols$Description,   mar = c(3.1, 3.1, 2.1, 10))


### Supplementary information

Supplementary Information

Supplementary_Table_1

## Data Availability

Code used to produce the LULC forecasts is available on our EnviDat repository (10.16904/envidat.458)^25^. The script is based on input data which are not included as they are not freely available, therefore it will not run as it is. These input data and their sources are listed in Supplementary Table 1; access to them can be requested to their owners. Alternatively, the code can be modified to run on other data.
