# Supplementary material for: High-resolution land use/cover forecasts for Switzerland in the 21st century
Source: Sci Data. 2024 Feb 23;11:231. doi: 10.1038/s41597-024-03055-z (PMC10891137; doi:10.1038/s41597-024-03055-z)
Supplement: Supplementary file 1 — Supplementary Information [file 41597_2024_3055_MOESM1_ESM.pdf]

Table of contents:

- Supplementary table 2: transition rules till the 2020-49 time-step.
- Supplementary table 3: transition rules for the 2045-74 and 2070-99 time-steps.

Supplementary table 2

	Industry	Building	Special urban	Urban green	Horticulture	Arable	Grassland	Alpine grassland	Forest	Brush	Trees	Unproductive vegetation	Bare land	Glacier
Industry	1	1	1	0	0	0	0	0	0	0	0	0	0	0
Building	0	1	0	0	0	0	0	0	0	0	0	0	0	0
Special urban	1	1	1	0	0	0	0	0	0	0	0	0	0	0
Urban green	1	1	1	1	0	0	0	0	0	0	0	0	0	0
Horticulture	0	0	0	0	1	0	0	0	0	0	0	0	0	0
Arable	1	1	1	1	1	1	1	0	0	0	0	0	0	0
Grassland	1	1	1	1	1	1	1	0	0	0	1	0	0	0
Alpine grassland	0	0	0	0	0	0	0	1	1	1	1	1	0	0
Forest	1	1	1	1	0	1	1	1	1	0	0	0	0	0
Brush	1	1	1	1	0	1	1	1	1	1	1	1	0	0
Trees	1	1	1	1	1	0	0	0	1	1	1	1	0	0
Unproductive vegetation	1	1	1	1	1	1	1	1	1	1	1	1	0	0
Bare land	0	0	0	0	0	0	0	0	0	0	0	1	1	0
Glacier	0	0	0	0	0	0	0	0	0	0	0	0	1	1

Supplementary table 3

	Industry	Building	Special urban	Urban green	Horticulture	Arable	Grassland	Alpine grassland	Forest	Brush	Trees	Unproductive vegetation	Bare land	Glacier
Industry	1	1	1	0	0	0	0	0	0	0	0	0	0	0
Building	0	1	0	0	0	0	0	0	0	0	0	0	0	0
Special urban	1	1	1	0	0	0	0	0	0	0	0	0	0	0
Urban green	1	1	1	1	0	0	0	0	0	0	0	0	0	0
Horticulture	0	0	0	0	1	0	0	0	0	0	0	0	0	0
Arable	0	0	0	0	1	1	1	0	0	0	0	0	0	0
Grassland	0	0	0	0	1	1	1	0	0	0	1	0	0	0
Alpine grassland	0	0	0	0	0	0	0	1	1	1	1	1	0	0
Forest	0	0	0	0	0	1	1	1	1	0	0	0	0	0
Brush	0	0	0	0	0	1	1	1	1	1	1	1	0	0
Trees	0	0	0	0	1	0	0	0	1	1	1	1	0	0
Unproductive vegetation	0	0	0	0	1	1	1	1	1	1	1	1	0	0
Bare land	0	0	0	0	0	0	0	0	0	0	0	1	1	0
Glacier	0	0	0	0	0	0	0	0	0	0	0	0	1	1
